# Iodide uptake by forest soils is principally related to the activity of extracellular oxidases

**DOI:** 10.3389/fchem.2023.1105641

**Published:** 2023-03-02

**Authors:** Russell M. Grandbois, Peter H. Santschi, Chen Xu, Joshua M. Mitchell, Daniel I. Kaplan, Chris M. Yeager

**Affiliations:** ^1^ Laboratory for Environmental and Oceanographic Research, Department of Marine Sciences, Texas A&M University—Galveston, Galveston, TX, United States; ^2^ Chemical Diagnostics and Engineering, Los Alamos National Laboratory, Los Alamos, NM, United States; ^3^ Savannah River Ecology Laboratory, University of Georgia, Aiken, SC, United States

**Keywords:** iodide, oxidase, forest soil, iodination, laccase, radioiodine, actinobacteria

## Abstract

^129^I is a nuclear fission decay product of concern because of its long half-life (16 Ma) and propensity to bioaccumulate. Microorganisms impact iodine mobility in soil systems by promoting iodination (covalent binding) of soil organic matter through processes that are not fully understood. Here, we examined iodide uptake by soils collected at two depths (0–10 and 10–20 cm) from 5 deciduous and coniferous forests in Japan and the United States. Autoclaved soils, and soils amended with an enzyme inhibitor (sodium azide) or an antibacterial agent (bronopol), bound significantly less ^125^I tracer (93%, 81%, 61% decrease, respectively) than the untreated control soils, confirming a microbial role in soil iodide uptake. Correlation analyses identified the strongest significant correlation between ^125^I uptake and three explanatory variables, actinobacteria soil biomass (*p* = 6.04E-04, 1.35E-02 for Kendall-Tau and regression analysis, respectively), soil nitrogen content (*p =* 4.86E-04, 4.24E-03), and soil oxidase enzyme activity at pH 7.0 using the substrate L-DOPA (*p* = 2.83E-03, 4.33E-04) and at pH 5.5 using the ABTS (*p* = 5.09E-03, 3.14E-03). Together, the results suggest that extracellular oxidases, primarily of bacterial origin, are the primary catalyst for soil iodination in aerobic, surface soils of deciduous and coniferous forests, and that soil N content may be indicative of the availability of binding sites for reactive iodine species.

## 1 Introduction

Radioiodine, a byproduct of nuclear power generation and weapons testing, poses a threat to human health due to its propensity to accumulate in the thyroid and its ability to release harmful beta (and weak gamma) particles. The significant environmental mobility of the reduced iodine species, iodide (I^−^), together with the 16-Myr half-life of ^129^I, makes remediation efforts to prevent the radionuclide from migrating outside of intended waste storage facilities critical to maintaining public health and safety.

Inorganic iodide can be immobilized in soils in the form of bound iodine. Charged iodine species can sorb to clays, hydrous oxides, and SOM can absorb I^−^, where sorption generally increases with decreasing pH (Whitehead, 1974; Shetaya et al., 2012; Miller et al., 2015). Most stable, soil-bound iodine in organic-rich soils is in the form of iodine covalently bound to organic matter (OM), particularly aromatic and N-bearing groups (I-org) (Schlegel et al., 2006; Franke and Kupsch, 2010; Xu et al., 2012; Xu et al., 2013). Formation of I-org in soils (i.e., soil iodination) is thought to proceed through a series of undefined, complex transfer mechanisms whereby reactive iodine intermediates, including molecular iodine (I_2_), hypoiodous acid (HOI), and triiodide, react with electron donating groups of OM (Rädlinger and Heumann, 2000; Reiller et al., 2006; Schlegel et al., 2006; Franke and Kupsch, 2010; Xu et al., 2012). The abiotic oxidation of I^−^ to I_2_ or HIO occurs very slowly due to I^−^ stability under pH and Eh conditions generally found in soil environments (Luther et al., 1995). However, multiple studies have shown that microbes and/or microbial enzymes can improve the kinetics of this reaction (Yeager et al., 2017a). Soil iodination has also been linked to microbial processes. Autoclaving, fumigation, air-drying, irradiation, heat treatments, and antibiotic applications have all shown to decrease soil iodination with I^−^ (Muramatsu et al., 1990; Muramatsu et al., 1996; Johanson, 2000; Amachi et al., 2003; Muramatsu et al., 2004), while addition of fresh soil was shown to restore iodination potential in autoclaved soils (Muramatsu et al., 2004). Soil iodination from iodide is also inhibited under anaerobic conditions, suggesting that oxidases, or other O_2_-dependent microbial processes, may be involved (Bors and Martens, 1992; Fukui et al., 1996; Bird and Schwartz, 1997).

Laccases are multi-copper oxidases produced by a wide range of organisms that perform a dizzying array of processes *via* one-electron oxidation of substrates (Janusz et al., 2020). While the most well-known source of laccase is wood-destroying white rot fungi, laccases (and laccase-like multi-copper oxidases) are also widespread among bacteria (Ausec et al., 2011; Bugg et al., 2011). In soil fungi the most notable function of laccase is to breakdown lignocellulose, while in bacteria their specific roles are often speculative and include morphogenesis and pigmentation, antibiotic production, spore protection, intercellular communication, lignocellulose degradation, etc., (Bugg et al., 2011; Janusz et al., 2020). While the redox potentials of laccases are often too low to directly oxidize the non-phenolic linkages of lignin (except for select high-redox-potential laccases of fungi), they can catalyze the formation of radicals in low molecular weight compounds, typically phenolics (e.g., vanillin, acetosyringone, p-coumaric acid, 4-hydroxybezylic alcohol, syringaldehyde, etc.), which then function as redox mediators that can oxidize linkages and depolymerize lignin (Bourbonnais et al., 1998; Camarero et al., 2005). Both fungal and bacterial laccases have been shown to oxidize halogens, including iodide (Christiansen Jesper and Carlsen, 1991; Xu, 1996; Suzuki et al., 2012). Though some bacterial laccases are capable of directly oxidizing I^−^, more commonly the process requires mediators (or at least is greatly enhanced in the presence of mediators), such as 2,2′-azino-bis(3-ethylbenzothiazoline-6-sulfonic acid) (ABTS) (Kulys et al., 2005; Ihssen et al., 2014; Shiroyama et al., 2015; Yeager et al., 2017a).

Previous studies have implicated (per)oxidases (mostly laccases) as catalysts of soil OM iodination under aerobic conditions *via* the oxidation of I^−^ (Shimamoto et al., 2011; Seki et al., 2013; Nihei et al., 2018). The fraction of iodine existing as organic iodine (org-I), measured by K-edge XANES and HPLC-ICP-MS, in depth profiles (0–12 cm) of iodine-rich soils in the Yoro area of Chiba, Japan were found to be correlated with laccase activity (Shimamoto et al., 2011). In the most comprehensive study to date examining the role of laccases in soil iodination, Seki et al. (2013) reported a strong linear correlation (*p* < 0.001) between partition coefficient (Kd) values for iodide and the specific activity of laccase in soils from two sites, rice paddy and forest soils, that were treated without or with a series of inhibitors (Seki et al., 2013). When they extended their analysis to a set of six, untreated Japanese soils (light-colored Andosol, gray lowland soil, sand-dune Regosol, dark red soil, humic Andosol, brown forest soil), a weak correlation (*R* ^2^ = 0.527; *p* = 0.102) was observed between laccase activity and I^−^ uptake. Addition of a bacterial iodide oxidase (laccase-type enzyme) to the forest and paddy soils strongly enhanced I^−^ uptake and partially restored I^−^ uptake in autoclaved soils. In a study from the same research group, it was found that fungal laccases could oxidize I^−^, but only in the presence of mediators (Nihei et al., 2018).

Overall, there is strong evidence that microbial laccases can stimulate soil iodination, but questions remain regarding the prevalence of this process and to what extent it contributes to soil iodination in natural soils. Additionally, the relative contribution of different microbial groups (e.g., fungal vs. bacterial) to soil iodination under aerobic conditions has not been determined. The aim of this study was to 1) confirm that soil oxidase activity contributes to ^125^I^−^ uptake under aerobic conditions in a collection of forest soils from Japan and the United States, 2) determine if “laccase activity could be used broadly as a proxy for iodide uptake capacity of different soils” (Seki et al., 2013), and 3) explore the relationship between pH, nitrogen and carbon soil content, and microbial biomass on biogenic soil iodination.

## 2 Materials and methods

### 2.1 Soil physiochemical and biomass analyses

A total of 20 soils were collected from forested sites in Japan and the United States at depths of 0–10 cm and 10–20 cm after removing surface debris and litter (Table 1). Field soils were frozen the day of collection, shipped on dry ice to Texas A&M University at Galveston (TAMUG), where they were immediately sieved (2 mm) and stored at −20°C for future analysis. Soil pH was measured using a bench Oakton^®^ pH/mv/°C meter (pH 510 series) in a 1:2.5 soil:water slurry. Soil dry weight was determined after 48 h drying at 105°C. Soil water holding capacity (WHC) was determined by saturating soil in a Buchman funnel for 30 min, allowing the soil to drain for 16 h and then drying the soil—the mass difference between wet and dry soils was used to calculate the WHC. Soil carbon and nitrogen content were measured using a Perkin Elmer 2400 Series II CHNS/O Analyzer after acidification with 1 M HCl (Ryba and Burgess, 2002). Soil biomass measures were determined by phospholipid fatty acid (PLFA) analysis carried out by MIDI Labs (Newark, DE).

**TABLE 1:** Soil metadata.

Sample	Depth^a^	Location^b^	Forest	pH	%C	%N	C/N
NTC10	0–10	NT	Coniferous	5.2	6.2	0.39	16.0
NTC20	10–20	NT	Coniferous	5.1	4.7	0.30	15.8
NTD10	0–10	NT	Deciduous	4.7	6.3	0.48	13.1
NTD20	10–20	NT	Deciduous	4.6	5.2	0.38	13.6
KAC10	0–10	KA	Coniferous	4.6	14.8	0.55	26.7
KAC20	10–20	KA	Coniferous	4.4	0.7	0.04	18.3
KAD10	0–10	KA	Deciduous	4.1	3.3	0.37	8.9
KAD20	10–20	KA	Deciduous	4.0	0.5	0.04	12.6
LAC10	0–10	LA	Coniferous	4.9	2.0	0.09	21.6
LAC20	10–20	LA	Coniferous	5.5	1.2	0.06	20.5
LAD10	0–10	LA	Deciduous	6.0	4.5	0.36	12.5
LAD20	10–20	LA	Deciduous	5.9	2.4	0.20	11.9
SRC10	0–10	SR	Coniferous	3.9	1.0	0.02	43.0
SRC20	10–20	SR	Coniferous	3.9	2.0	0.04	45.8
SRD10	0–10	SR	Deciduous	4.4	7.5	0.44	17.1
SRD20	10–20	SR	Deciduous	4.3	6.3	0.33	19.0
FUC10	0–10	FU	Coniferous	5.7	21.2	1.16	18.2
FUC20	10–20	FU	Coniferous	5.9	3.0	0.23	13.0
FUD10	0–10	FU	Deciduous	5.9	9.7	0.75	12.9
FUD20	10–20	FU	Deciduous	6.0	2.0	0.22	9.2

^a^ Soils were collected from 0 to 10 cm or 10–20 cm below the surface after debris and litter had been brushed aside.

^b^ Location abbreviations: NT, Nishi-Tokyo, Japan; KA, Katano, Osaka, Japan; LA, Los Alamos, NM, United States.; SR, Savannah River Site, SC, United States; FU, Fukushima Prefecture, Japan.

### 2.2 Oxidase enzyme activities

To quantify oxidase activity in soils, we adapted the procedure described by Bach et al. (2013). Soil (0.5 g) was added to a 100-mL solution of either 50 mM sodium acetate buffer (pH 5.5) or 25 mM maleic acid buffer (pH 7) and homogenized using a Waring blender at 20,000 rpm for 1 min. Triplicate soil suspensions (200 µL) were then transferred to a 96-well plate. Assays were initiated by the addition of 50 µL substrate (2 mM ABTS for assays conducted at pH 5.5; 25 mM L-DOPA for pH 7 assays) to each well. Absorbance was measured every 15 min at 420 nm (ABTS) and 460 nm (L-DOPA) over 2.5 h at 25°C. Absorbance values of control wells containing slurry without substrate and substrate without slurry were subtracted from the slurry-substrate wells. Using the extinction coefficients of 42 for ABTS (pH 5.5) and 8.9 for L-DOPA (pH 7), soil oxidase activities (µmol h^−1^ g^−1^ soil) were calculated using the following equation:

$$\left(NetAbs\;\times \;100\;mL\;\left(soil\;in\;buffer\right)\right)/\left(ExtCoeff\;\left(µmol\right)\times \;0.2\;mL\;\left(sample\;in\;well\right)\;\times \;time\;\left(h\right)\times \;dry\;wt\;soil\;in\;buffer\;\left(g\right)\right)$$

### 2.3 ^125^I soil uptake

To determine the iodine sorption capacity of soils, 0.5 g of freshly thawed or autoclaved soils were added to 100 mL of artificial freshwater (pH 7) (Xu et al., 2011) and homogenized in a Waring blender at 20,000 rpm for 1 min. Samples (1 mL) of the resulting soil slurry were transferred, in triplicate, to 1.5-mL microcentrifuge tubes and incubated in the dark for 12 h at 25°C with shaking at 150 rpm. The effect of various antimicrobials and enzyme inhibitors on ^125^I uptake was tested by amending fresh soil slurries with either 3 g·L^−1^ cycloheximide (antifungal), 3 g·L^−1^ bronopol (bacterial and fungal biocide), or 10 mM sodium azide (oxidase and respiration inhibitor) during the 12 h incubation period. After the 12-h incubation, 2 × 10^9^ cpm/L sodium iodide (^125^I) was added to each soil slurry and the tubes were incubated under the same conditions for 24 h. The soil slurries were then centrifuged at 15,000 x g for 15 min and a portion of the supernatant (100 µL) was removed to measure ^125^I activity (cpm) using a Beckman Coulter Liquid Scintillation Counter (Model No: LS6500). Percent ^125^I uptake by soils was calculated by dividing the ^125^I sorbed to the soil (calculated as the difference between the total ^125^I amended initially and the residual ^125^I in the supernatant after the incubation) to the total ^125^I activity amended initially.

### 2.4 Statistical analyses

Correlation coefficients between soil properties were calculated using the Kendall-Tau rank correlation coefficient. Differences in soil properties with respect to depth and forest type were examined using the Mann-Whitney *U* test. Possible effects of sampling site on soil properties were explored using the Kruskal-Wallis form of one-way ANOVA. Linear regression models were constructed for all unique pairs of soil properties and multiple linear regression models constructed for most permutations of potentially explanatory properties. Finally, 2-component Principal Component Analysis (PCA) was performed using all soil properties and iodide uptake after standardizing all feature values to a mean of 0 and unit variance. Mean iodide and oxidase activity values for each sample (*n* = 3) were used for all analyses and categorial variables were dropped prior to PCA analysis. Values used for statistical analysis are provided in Supplementary Table S1.

Multiple testing correction was performed to minimize the likelihood of false positives. Correction of *p*-values for correlation, linear regression and multiple regression analyses was performed using the Bonferroni method (Gelman et al., 2012; Ranstam, 2016), which can yield adjusted *p*-values greater than 1.0, while all other analyses were corrected using the Benjamini-Hochberg method (Benjamini and Hochberg, 1995). Comparisons of soil properties with respect to sampling site, depth, and forest type were considered as independent sets of tests for the purposes of multiple testing correction. No pairing of samples was considered for any analysis given our desire to detect differences that are distinct from depth or forest-type variance within a single sampling site. Only adjusted *p*-values are reported and an adjusted *p*-value cutoff of 0.05 was used for determining statistical significance while adjusted *p*-values between 0.05 and 0.10 were considered “near-significant.”

General data processing was performed using Python version 3.9.10 (Van Rossum and Drake Jr, 1995), Numpy version 1.23.4 (Harris et al., 2020), and Pandas version 1.5.1 (McKinney, 2010; The pandas development team, 2022). All statistical tests, except multiple regression (Microsoft^®^ Excel for Mac Version 16.67), were performed using their implementation in Scipy version 1.9.3 (Virtanen et al., 2020) and PCA was performed using the pca library version 0.1.0 (Erdogan, 2020) with feature standardization performed using the StandardScaler from Sklearn version 1.1.3 (Pedregosa et al., 2011). Benjamini-Hochberg correction was performed using the multipletests function in statsmodels version 0.13.2 (Seabold and Perktold, 2010) while Bonferroni correction was performed using a custom routine. Visualization was performed using matplotlib version 3.6.0 (Hunter, 2007) and Seaborn version 0.12.1 (Waskom, 2021).

## 3 Results

### 3.1 Soil chemical properties

Twenty soil samples (0–10 cm and 10–20 cm depths) collected from deciduous and coniferous zones of 5 forested sites in Japan and the United States (Location abbreviations: NT—Nishi-Tokyo, Japan; KA—Katano, Osaka, Japan; LA—Los Alamos, NM, United States; SR—Savannah River Site, SC, United States; FU—Fukushima Prefecture, Japan) were analyzed for pH, %C, %N and biomass (PLFA) (Table 1). Soils were acidic (mean, 4.95), and pH varied significantly between sites (*p* = 0.037). SR soils had the lowest mean pH (4.13) while FU soils had the highest mean pH (5.88). Across all sites, soil pH did not vary as a function of forest type (coniferous, pH 4.9; deciduous, pH 5.0; *p =* 0*.*791) or depth (0–10 cm, pH 4.9; 10–20 cm, pH 4.9; *p* = 0.970).

Although sampling location was not a significant factor for either C or N content (*p =* 0*.*715 and *p =* 0*.*684, respectively) a range of values were observed for these properties across samples. The mean C and N content of the soils were 5.2% and 0.32% respectively with FU soils possessing both the highest C (9.0%) and N content (0.60%), while LA samples exhibited the lowest mean C (2.5%) and SR samples the lowest mean N content (0.18%). N content was nearly significantly different between soil depths (0–10 cm 0.46%; 0–20 cm 0.18%; *p* = 0.063); as was C content (0–10 cm 7.65%; 0–20 cm 2.8%; *p* = 0.063). There was not a significant effect of forest type on C or N content (*p =* 0*.*613 and *p =* 0*.*442 respectively). The C/N ratio, which averaged 18.5 across all samples, was significantly higher in the coniferous soils than deciduous soils (means 23.9 and 13.1, respectively, *p =* 0*.*002). The SR coniferous soils collected at both depths exhibited notably high C/N ratios (43.1 for 0–10 cm; 45.8 for 10–20 cm).

### 3.2 Soil biomass

The twenty soils were analyzed for biomass content using PLFA and averaged 123.8 nmol total PLFA per g soil, ranging from 99 nmol·g^−1^ in SR soils to 151 nmol·g^−1^ in NT soils (Table 2). As an example of extremes, SR coniferous soils samples collected from 0–10 cm and 10–20 cm contained an average of 24.4 and 33.6 nmol total PLFA g^−1^ soil, respectively, while NT coniferous soils exhibited 257.8 and 50.9 nmol total PLFA g^−1^ soil in the 0–10 cm and 10–20 cm samples, respectively. Bacterial PFLAs accounted for 89.1%–96.3% of total biomass, fungal PFLAs accounted for 3.7%–9.3% of total biomass and eukaryotic content less than 2.1% of total biomass. Although a wide range of gram-positive and gram-negative content was observed in our samples [gram (+): 11.1–81.7 nmol/g; gram (−): 8.4–120.7 nmol/g], all but three samples (SR coniferous 0–10 cm, SR coniferous 10–20 cm, and FU deciduous 10–20 cm) displayed a higher abundance of gram (+) biomass than gram (−). *Actinomyces* accounted for 10.5%–21.7% of total bacterial biomass, with NT and FU deciduous forest soils having the highest concentrations.

**TABLE 2:** Phospholipid fatty acid content of soil^a^.

Sample^b^	Total^c^	Bacteria	Fungi	Bacterial taxa		
				Gram (−)	Gram (+)	Actinobacteria
NTC10	156.3	145.5	9.0	65.9	51.5	28.1
NTC20	78.7	74.5	3.5	31.9	25.8	16.8
NTD10	197.4	186.9	8.7	97.7	59.8	29.3
NTD20	172.7	164.1	6.6	81.4	52.6	30.1
KAC10	257.8	229.7	24.1	120.7	81.7	27.2
KAC20	50.9	48.0	2.4	22.1	18.2	7.7
KAD10	157.3	143.0	11.1	65.9	60.6	16.5
KAD20	45.6	42.9	2.2	18.8	17.2	6.9
LAC10	79.2	71.1	6.5	33.5	28.3	9.3
LAC20	42.6	40.4	1.7	18.5	13.9	7.9
LAD10	170.4	153.3	13.8	81.5	53.7	18.0
LAD20	125.3	116.3	6.4	55.3	43.3	17.7
SRC10	24.4	23.5	0.9	8.4	11.1	4.0
SRC20	33.6	32.0	1.5	12.2	14.3	5.4
SRD10	209.8	196.1	12.4	105.4	68.3	22.4
SRD20	127.1	120.0	6.4	64.4	40.9	14.7
FUC10	126.1	118.0	7.0	59.9	38.7	19.3
FUC20	92.6	87.8	3.9	37.2	32.8	17.8
FUD10	241.3	227.8	10.3	115.5	73.9	38.4
FUD20	86.2	82.0	3.2	30.7	32.6	18.7

^a^ Values are reported as nmol of phospholipid fatty acids extracted per g of soil (dry weight).

^b^ Sample abbreviations are the same as in Table 1.

^c^ Total includes eukaryotic PLFA biomass, which is not shown.

All soils displayed greater total biomass at surface to 10 cm depth compared to the same soil’s 10–20 cm depth, except for the SR coniferous soils. All classes of PLFAs were greater in the 0–10 cm soil samples than those collected from 10–20 cm below the surface; however, only fungi biomass was significantly different between depths (0–10 cm 10.373 nmol/g; 0–20 cm 3.771 nmol/g, *p* = 0.047). Nearly all other PFLAs measurements [i.e., total biomass, gram (−), gram (+), and total bacteria] were near-significant with respect to soil depth (*p* ≥ 0.063). Notably, *Actinobacteria* biomass was not significantly different between soil depths (*p* = 0.150) and sample site was not associated with any biomass measurement (*p* ≥ 0.196).

### 3.3 Soil oxidase activity

Scoping experiments were performed to determine the optimal pH for ABTS and L-DOPA oxidation across the forest soil samples. For ABTS pH values of 4.0 and 5.5 were evaluated, and for L-DOPA we tested oxidation rates at pH 7.0 and 8.0. Additionally, 0.3% hydrogen peroxide was added to soil/ABTS or L-DOPA slurries to determine peroxidase activity. Based on these experiments (data not shown) soil oxidase activity was measured using the substrates ABTS and L-DOPA at pH 5.5 and pH 7, respectively. Addition of hydrogen peroxide did not stimulate additional ABTS activity for most soil samples tested and only resulted in a slight increase (<10%) for L-DOPA oxidation rates in ∼50% of the samples. Because peroxidase activity was detected sporadically amongst the soil samples, and, when detected, it contributed little to the overall rates of ABTS or L-DOPA oxidation, we did not further examine peroxidase activity in this study.

**FIGURE 1:**
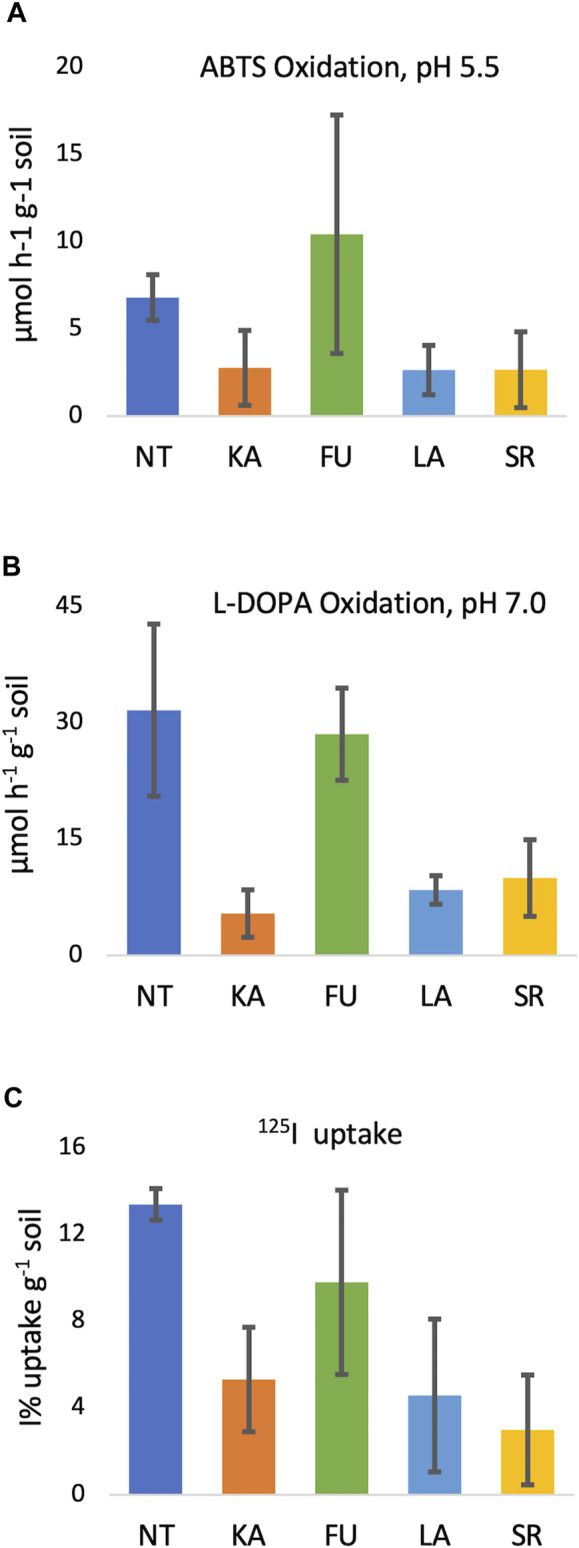
Extracellular oxidase activity and ^125^I uptake in soils. **(A)** ABTS oxidation (µmol h^−1^ g^−1^ soil) at pH 5.5, **(B)** L-DOPA oxidation (µmol h^−1^ g^−1^ soil) at pH 7.0; **(C)** ^125^I uptake (%I uptake per mg dry soil, relative units).

With both substrates (ABTS and L-DOPA at pH 5.5 and pH 7, respectively), soils from sites KA, LA, and SR exhibited less oxidase activity than soils from sites NT and FU (Table 3). The difference was particularly pronounced using L-DOPA, where mean oxidation rates ranged from 5–10 μmol·h^−1^·g^−1^ soil versus 29–32 μmol·h^−1^·g^−1^ for the NT and FU soils. These findings reflect that sampling site is significantly associated with oxidase activity at pH 7 with L-DOPA (*p* = 0.039) and near significantly associated with oxidase activity at pH 5.5 (*p =* 0.069) (Figure 1). Soil oxidase activity did not correlate with soil depth or forest type (coniferous vs. deciduous).

**TABLE 3:** Soil oxidase activity^a^.

Sample^b^	ABTS (pH 5.5)	L-DOPA (pH 7.0)
NTC10	8.1 ± 0.1	19.4 ± 7.4
NTC20	5.8 ± 0.1	48.0 ± 2.5
NTD10	7.9 ± 1.0	31.4 ± 3.4
NTD20	5.3 ± 0.7	27.7 ± 2.8
KAC10	5.8 ± 3.6	8.3 ± 1.2
KAC20	0.8 ± 0.1	2.2 ± 1.7
KAD10	3.2 ± 0.4	8.2 ± 3.2
KAD20	1.2 ± 0.1	2.9 ± 1.9
LAC10	2.5 ± 0.5	8.0 ± 0.3
LAC20	0.6 ± 0.2	6.0 ± 1.4
LAD10	3.2 ± 0.3	9.0 ± 2.7
LAD20	4.2 ± 0.8	10.8 ± 2.1
SRC10	0.1 ± 0.1	3.8 ± 1.7
SRC20	0.8 ± 0.5	7.4 ± 2.3
SRD10	2.9 ± 1.0	13.5 ± 1.2
SRD20	5.0 ± 0.3	15.3 ± 1.6
FUC10	21.0 ± 0.4	33.3 ± 4.2
FUC20	8.5 ± 0.6	24.3 ± 2.4
FUD10	8.5 ± 2.2	34.7 ± 8.3
FUD20	3.7 ± 1.0	21.9 ± 1.3

^a^ Values are reported as µmol of substrate oxidized per hour per g soil (dry weight) ± standard deviation (3 replicate measurements) rounded to one decimal place.

^b^ Sample abbreviations are the same as shown in Table 1.

### 3.4 ^125^I soil uptake

Iodine binding capacity of the soils was assessed with or without several inhibitors of biological activity (i.e., cycloheximide, antifungal; bronopol, bacterial and fungal biocide; sodium azide, oxidase and respiration inhibitor; autoclaving, kills cells/inactivates enzymes) (Table 4). While each untreated soil displayed some ability to take-up the ^125^I^−^ tracer, they did so to varying degrees (1.0%–16.4% ^125^I uptake; mean = 7.2%), with a near-significant association with sampling site (*p* = 0.069) (Figure 1). On average, NT soils exhibited the highest %^125^I^−^ uptake (12.5%–14.4%). ^125^I uptake was also pronounced in the FU 0–10 cm surface soils (16.4% and 11.5% ^125^I uptake for coniferous and deciduous forest soils, respectively), and was >2 fold greater than uptake in their paired 0–20 cm samples (6.1% and 5.3%). SR coniferous soils sorbed >10 fold less ^125^I^−^ (1%–1.3% ^125^I^−^ uptake) than the NT and FU surface soils. Notably, ^125^I uptake was not correlated with soil depth (*p* = 0.150) or forest type (*p =* 0*.*791) across all samples.

**TABLE 4:** ^125^I uptake activity and effect of inhibitors in forest soils.

Sample^a^	Fresh soil^b^	Inhibitor treatment^c^			
		Autoclave (%)	Cycloheximide (%)	Bronopol (%)	Sodium azide (%)
NTC10	13.3 ± 0.7	2.9	112.3	92.8	34.4
NTC20	14.4 ± 0.6	1.8	99.1	76.0	19.4
NTD10	13.4 ± 0.1	1.9	100.1	67.3	30.2
NTD20	12.5 ± 0.3	1.8	106.6	76.4	24.0
KAC10	8.5 ± 0.7	2.5	97.6	42.9	11.9
KAC20	3.4 ± 0.4	6.3	54.0	17.0	17.3
KAD10	6.7 ± 0.4	4.0	93.2	26.6	7.0
KAD20	2.7 ± 0.2	3.3	79.9	25.2	15.3
LAC10	4.5 ± 0.3	8.5	84.3	14.8	0.9
LAC20	3.0 ± 0.2	6.0	95.0	19.6	1.0
LAD10	6.1 ± 0.4	3.3	101.5	33.1	12.1
LAD20	4.7 ± 0.2	5.5	108.5	36.0	18.9
SRC10	1.0 ± 0.1	7.1	9.1	11.1	14.1
SRC20	1.3 ± 0.1	2.4	31.0	18.3	16.7
SRD10	6.2 ± 0.9	1.1	116.8	64.7	5.2
SRD20	3.5 ± 0.7	0.6	113.0	22.7	7.4
FUC10	16.4 ± 1.9	21.0	90.9	33.2	39.7
FUC20	6.1 ± 0.2	12.1	123.5	39.8	45.4
FUD10	11.5 ± 1.2	15.3	104.5	35.1	35.1
FUD20	5.3 ± 0.1	16.0	105.5	24.0	21.1

^a^ Sample abbreviations are the same as shown in Table 1.

^b^ Values are reported as % ^125^I uptake per mg of dry soil ± standard deviation (3 replicate measurements) rounded to one decimal place.

^c^ Values are mean percent ^125^I uptake activity relative to fresh soils when treated with listed inhibitors.

Biological inhibitors affected the different soils’ abilities to take-up ^125^I to varying degrees, with some inhibitors showing no inhibition of ^125^I uptake while other showed nearly complete inhibition of ^125^I^−^ uptake, and some enhancing ^125^I^−^ uptake (Table 4). The antifungal agent, cycloheximide, exhibited the least inhibitory effect on ^125^I^−^ uptake. Except for four soils (SRC10, 9%; SRC20, 31%; KAC20, 54%; KAD20, 80%), cycloheximide treatment resulted in <25% change (i.e., inhibitory effect as compared to the fresh soil without antifungal agent) in ^125^I uptake. Interestingly, cycloheximide displayed a stimulatory effect on ^125^I^−^ uptake in half the soils tested.

Applications of the antimicrobial agent, bronopol, and the oxidase and respiration inhibitor, sodium azide (NaN_3_), each resulted in significant inhibition of ^125^I^−^ uptake compared to fresh soil controls, while autoclaving soils resulted in nearly complete inhibition of ^125^I^−^ uptake (Table 4). Bronopol inhibition ranged from 7%–89% with an average inhibition of 61%; NaN_3_ inhibition ranged from 55%–99% with an average inhibition of 81%; and autoclaving inhibited ^125^I^−^ uptake from 79%–99% with an average inhibition of 94%. KA, LA, and SR soils responded remarkably similarly to the inhibitors, with inhibition ranges of 94%–97%, 71%–74% and 87%–92% for autoclaving, bronopol and sodium azide treatments, respectively. The NT soils exhibited a similar level of inhibition from autoclaving (98%), but slightly lower inhibition from sodium azide (73%) and much lower inhibition when using bronopol (22%). The inhibitors were generally less effective in FU soils, with autoclaving, bronopol and sodium azide resulting in 84%, 67%, 65% inhibition of ^125^I uptake, respectively. Overall, these results provide clear evidence for the dominant role of biological processes in ^125^I^−^ uptake among deciduous and coniferous forest soils, up to 20 cm depth, from two continents.

### 3.5 Correlation of ^125^I uptake with soil properties, biomass, and extracellular enzyme activity

#### 3.5.1 Kendall-Tau rank correlation

**FIGURE 2:**
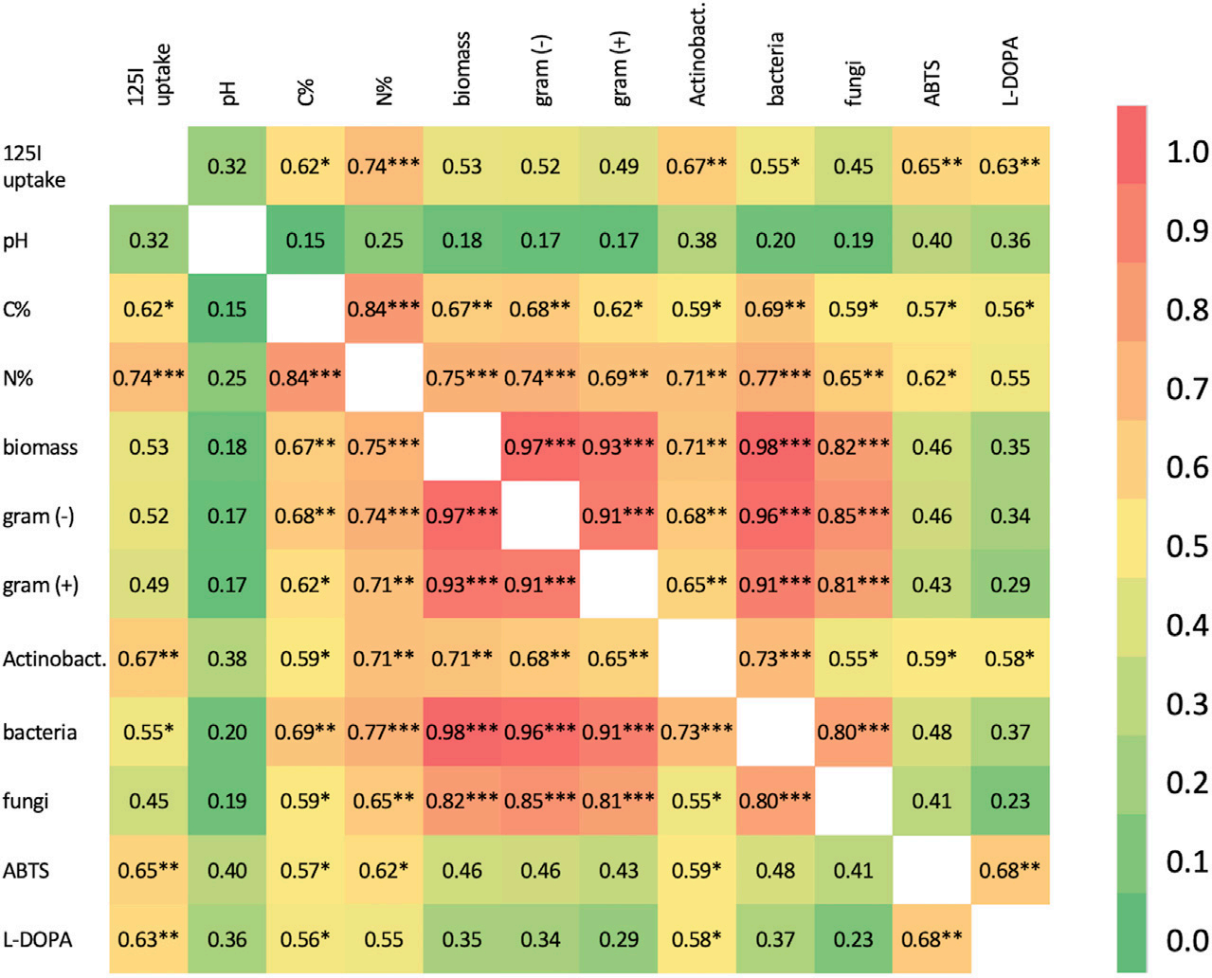
Kendall Tau correlation matrix for measured properties, except C/N ratio, which is negatively correlated with nearly all soil properties, across all soil samples. Correlations are marked significant at the 0.05*, 0.01** and 0.001*** levels (Supplementary Table S2 for raw data). Actinobact, Actinobacteria.

Kendall-Tau rank correlation was used to investigate the intersection between measured soil properties, biomass, extracellular oxidase activity and ^125^I^−^ uptake (Figure 2). Most soil property pairs exhibited some degree of positive correlation, except for the C/N ratio which was negatively correlated with most other properties (not shown, see Supplementary Table S2), most of these correlations are not significant after multiple testing correction and when disregarding comparisons between obviously linked properties (e.g., bacterial biomass vs. total biomass, C% and biomass, etc.). With regards to ^125^I^−^ uptake, it was most strongly correlated with N content (*τ* = .74, *p* = 4.86E-04), followed closely by actinobacterial biomass (*τ* = .67, *p* = 6.04E-04) and ABTS and L-DOPA oxidase activities (*τ* = .65 and .63; *p* = 5.09E-03, 2.83E-03, respectively). A moderate correlation with C content (*τ* = .62, *p* = 1.26E-02) and bacterial biomass (*τ* = .55, *p* = 3.75E-02) was also observed for ^125^I^−^ uptake.

The strong correlation between *Actinomyces* spp. biomass and ^125^I^−^ uptake is quite striking. A strong block diagonal correlation structure is observed among total biomass, gram (+) biomass, gram (−) biomass and bacterial biomass with τ′s > .91 and highly significant corrected *p*-values. Interestingly, although *Actinomyces* biomass is correlated with these other bacterial biomass measurements, these correlations are relatively weak by comparison with the largest *τ* of .73 observed between total bacterial and *Actinomyces* biomass. Additionally, the other measurements of bacterial subpopulation biomass are not significantly correlated with iodide uptake suggesting that *Actinomyces* contributes to this process in a manner distinct from other bacterial subpopulations.

Oxidase activity was also significantly correlated with *Actinomyces* biomass (pH 5.5, *τ* = .59, *p =* .0254; pH 7, *τ* = .58, *p* = .0152) as well as C content (pH 5.5, *τ* = .58, *p =* .0270); however, deciphering if this is a direct or indirect correlation is difficult given the significant correlations between *Actinomyces* biomass, C content and N content without orthogonal information.

#### 3.5.2 Regression analysis

**FIGURE 3:**
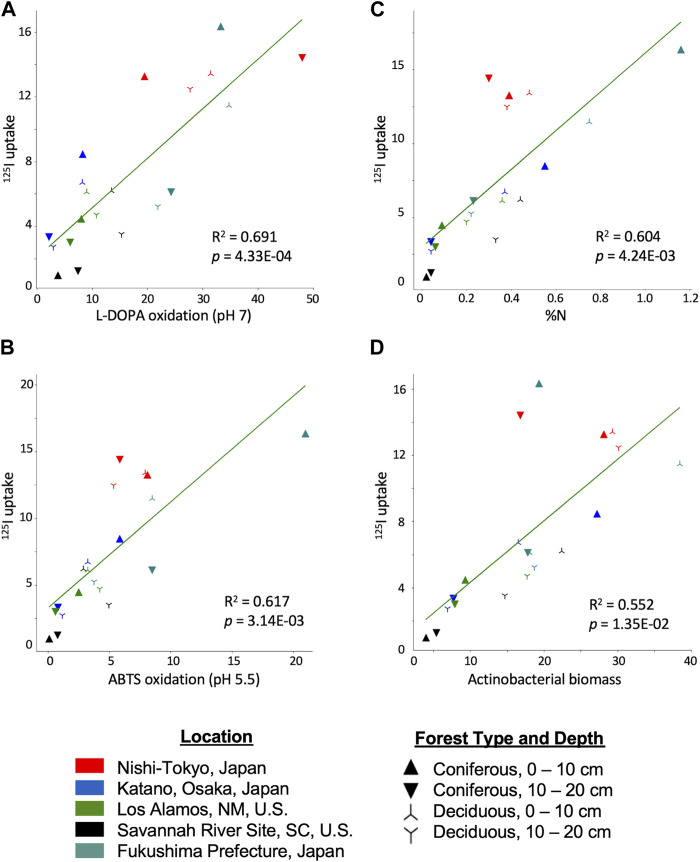
^125^I uptake from forest soils correlated with **(A)** L-DOPA oxidation (pH 7.0), **(B)** ABTS oxidation (pH 5.5), **(C)** soil %N, **(D)** actinobacterial biomass. Oxidase activity units are reported as μmol h^−1^ g^−1^ dry soil. Actinobacterial biomass reported as nmol PLFAs g^−1^ dry soil. Site locations are designated by color: NT, red; KA, blue; LA, green; SR, black; FU, teal. Coniferous 0–10 cm, right side up triangle; Coniferous 10–20 cm, upside down triangle; Deciduous 0–10 cm, right side up star; Deciduous 10–20 cm, upside down star.

Linear regression was performed between all soil property pairs which revealed multiple factors that exhibit some degree of a linear relationship with ^125^I^−^ uptake. Oxidase activity at pH 7.0 yielded the highest explained variance with an *R* ^2^ of .691 (*p =* 4.33E-04) followed by oxidase activity at pH 5.5 (*R* ^2^ = .617, *p* = 3.14E-3), N content (*R* ^2^ = .604, *p* = 4.24E-03), and *Actinobacteria* biomass (*R* ^2^ = .552, *p* = 1.35E-02) (Figure 3; panels A, B, C and D, respectively). Although the rank order of the *R* ^2^ values does not perfectly correspond to the rank order of the Kendall-Tau correlations, the overall patterns remain similar. Notably, the NT samples consistently had higher than expected ^125^I^−^ uptake than expected by the regression models. Because the NT samples were outliers, linear regression was performed without these samples (Supplementary Figure S1). *R* ^2^ values decreased somewhat for the correlation between ^125^I^−^ uptake and oxidase activity measured with L-DOPA at pH 7.0 (*R* ^2^ = .616, *p* = 0.024) or actinobacterial biomass (*R* ^2^ = .526, *p* = .116). In contrast, the *R* ^2^ increased for the correlation between ^125^I^−^ uptake and oxidase activity measured with ABTS at pH 5.5 (*R* ^2^ = .824, *p* = 9.27E-05) and the correlation with soil %N was exceptionally strong (*R* ^2^ = .930, *p* = 1.37E-07). In comparison, the *R* ^2^ for the correlation between soil %C and ^125^I^−^ uptake when using the dataset minus the NT samples was 0.775.

Multiple regression analysis of permutations of the potential explanatory variables was also performed (Supplementary Table S3). The results highlighted the importance of the measure of oxidase activity, particularly using L-DOPA at pH 7.0, as the primary explanatory variable with the full data set. When oxidase activity at pH 7.0 was combined with one of the other top predictor variables (e.g., C or N content, total biomass, actinobacterial biomass, oxidase activity measured at pH 5.5 with ABTS), multiple regression analysis yielded an adjusted *R* ^2^ of 0.75 or higher (an adjusted *R* ^2^ 0.797, with oxidase activity at pH 7.0 and %N as the explanatory variables, was the highest two-factor correlation). Addition of other variables measured in this study did not substantially increase the goodness-of-fit with ^125^I^−^ uptake. The highest adjusted *R* ^2^ value obtained using 4 variables (oxidase activity at pH 7.0, oxidase activity at pH 5.5, actinobacterial biomass, pH) was 0.826 and the highest adjusted *R* ^2^ was 0.857 with 9 variables (Supplementary Table S3). When the NT samples are excluded from the analysis, soil %N provided 93% of the explanatory power, as discussed above. Including either L-DOPA, ABTS or actinobacterial biomass measures with the %N data did not result in a better fit; with all four variables a *R* ^2^ of 0.953 (adjusted *R* ^2^ 0.936) was obtained.

#### 3.5.3 PCA analysis

**FIGURE 4:**
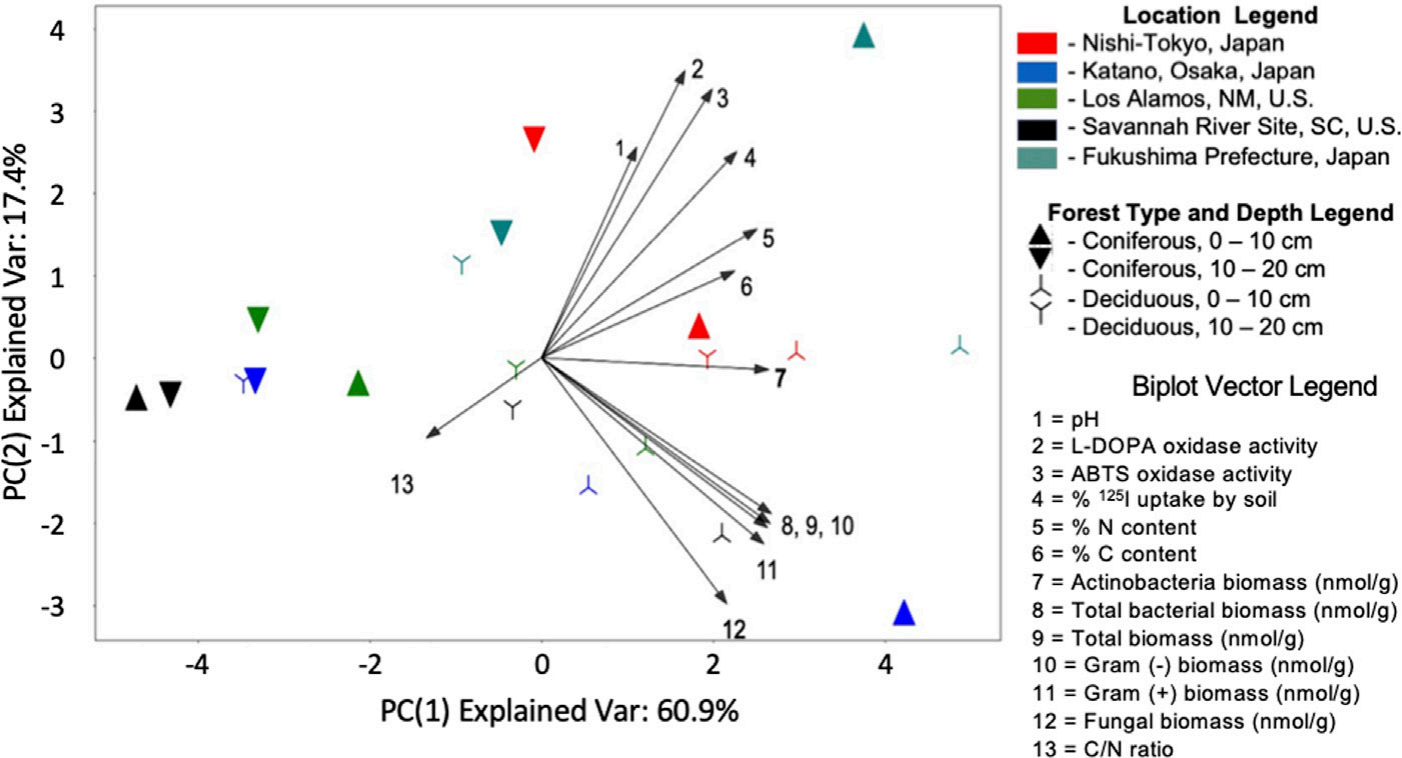
Actinobacteria biomass contributes to sample variance in a distinct manner. Two-component principal component analysis was performed on the standardized, non-redundant, numerical features of the samples. The resulting model explained 78.4% of variance in the dataset, with the majority of the explained variance contained within principal component 1. Samples with high iodine uptake aggregate in the upper right corner while samples from coniferous forests tend to aggregate towards the left side of the plot along PC1. The contributions of each property to the PCA model is represented in the biplot. Highly correlated features result in near-parallel vectors as seen with the non-Actinomyces biomass vectors (vectors 8–12) and chemical properties vectors (vectors 1–6) clustering together. *Actinomyces* biomass, vector 7, contributes almost exclusively to the most informative principal component and does not cluster with any other property. Similarly, the C/N ratio vector (vector 13) is distinct and may explain the aggregation of coniferous samples towards the left of the plot.

2-Component PCA was performed using the standardized values of all numerical properties (Figure 4). The two-component model explained 78.3% of the dataset variance with the majority explained by PC1 (60.9%). Samples with high iodine uptake (NT and FU 0–10 cm) aggregate to the upper right corner of the plot.

Highly correlated soil properties yield near-parallel vectors in the biplot. The biomass measurements (except *Actinobacteria* biomass) cluster towards the bottom right of the plot (vectors 8–12) while iodine uptake, oxidase activity and soil nitrogen and carbon content cluster together towards the top right. Notably, *Actinobacteria* biomass (vector 7, in bold) contributes almost exclusively to the most informative principal component, PC1. This finding, combined with our previous analyses, supports the hypothesis that *Actinobacteria* biomass contributes in distinct manner to the variance observed between these soil samples. These trends remain largely unchanged when the NT samples are dropped from the analysis (Supplementary Figure S2).

## 5 Discussion

Both fungi and bacteria have been shown to be capable of iodide-oxidation (Yeager et al., 2017a; Nihei et al., 2018). In the current study, the weak inhibitory effects of cycloheximide treatment, low overall percentage of fungal biomass (mean, 5.3%), and poor correlation between fungal biomass and ^125^I^−^ uptake (*τ* = 0.45, *p* = 0.37) indicated a relatively minor role for fungi in ^125^I^−^ uptake compared to bacteria in temperate forest soils. The four soils in which cycloheximide exhibited a notable inhibitory effect (i.e., >20% reduction in ^125^I^−^ uptake) were also the soils with the lowest N content (0.02–0.04 %N) and low overall biomass. Though the percent fungal biomass among these samples (3.6%–4.7%) was slightly lower than average across all sites, it is possible that fungi play a larger role in soil iodination when N is extremely limiting, excreting (per)oxidases to harvest N from difficult to degrade substrates such as lignin (Wymelenberg et al., 2009; Zavarzina et al., 2018). It is important to emphasize that the litter/humus layer, where fungal activity dominates in forest ecosystems, was removed from forest soils prior to collection in the current study. To our knowledge, the extent to which fungal activity influences iodine speciation within the litter layer has not been determined, though org-I is found in this top level of the forest floor (Roulier et al., 2019). Nor is it known (and our data cannot distinguish) if fungal laccases secreted in the litter layer can percolate into the upper soil layer and contribute to iodination.

The results presented here indicate that actinobacteria may play an oversized role in soil iodination in some forest soils. First, ^125^I^−^ uptake was strongly correlated with actinobacterial biomass, but not with other subpopulations [i.e., gram (+), gram (−), fungi] of microbial biomass (Figure 2). Second, actinobacterial biomass was the only PLFA biomass measure with a significant Kendall-Tau correlation with oxidase activity (ABTS and L-DOPA) (Figure 2). Third, PCA analysis showed that the actinobacteria biomass acts distinctly with regards to soil properties, ^125^I^−^ uptake and oxidase activity relative to the other measures of biomass (Figure 4). Fourth, amongst the measures of microbial biomass determined, actinobacterial biomass was the only that, when combined with other explanatory variables (e.g., %N, %C, pH, oxidase activity), consistently improved goodness-of-fit for multiple regression analysis of ^125^I^−^ uptake (Supplementary Table S3). Finally, it was observed that soils with the highest actinobacterial biomass, NT soils, also had the highest mean ^125^I^−^ uptake, and for each of the other soils (FU, KA, LA, SR), the sample with the highest iodide uptake was also the inter-site sample with the largest *Actinomyces* content.

Members of the Actinobacteria are cosmopolitan, inhabiting most ecological niches and are well known for their ability to degrade complex polymers (e.g., lignin, cellulose, etc.) in litter and soils (Štursová et al., 2012; Yeager et al., 2017b). Along with typical three-domain laccases common in fungi, plants and insects, bacterial also produce laccase-like multicopper oxidases with two structural domains (Janusz et al., 2020). A unique class of these two-domain bacterial laccases, SLACs (Small LACase), were first discovered and are common in the Actinobacteria (Machczynski et al., 2004) (Fernandes et al., 2014). SLACs exhibit several unique properties that could set them apart with regards to I^−^ oxidation in soils. First, unlike some bacterial laccases, which are mostly present intracellularly, SLACs are typically secreted and serve as extracellular enzymes (Dubé et al., 2008). Second, they are active over an unusually high pH range (mildly acidic to basic; fungal laccases are typically optimal at pH 3.0–5.5) relative to other laccases (Janusz et al., 2020). Third, they exhibit high thermo- and salt-tolerance and are considered more resistant to denaturation than typical laccases (Fernandes et al., 2014). Fourth, some are relatively resistant to inhibitors, including sodium azide (Trubitsina et al., 2015) (Fernandes et al., 2014). To our knowledge actinobacterial SLACs have not been evaluated for I^−^ oxidation. It is, however, notable that the two well-characterized bacterial iodide oxidases, IoxA from *Roseobacter* strain Q-1and *Roseovarius* strain A-2, both share these traits (except for sodium azide resistance) (Suzuki et al., 2012; Shiroyama et al., 2015).

The soils could be divided into two sets with regards to oxidase activity, ^125^I^−^ uptake activity, inhibition patterns and actinobacterial biomass. Soils from sites KA, LA and SR contained relatively low actinobacterial biomass (mean, 12–15 nmol actinobacterial PLFA g^−1^ soil; 13%–14% of the bacterial biomass) compared to soils from sites NT and FU (mean, 24–26 nmol actinobacterial PLFA g^−1^ soil; 18% of the bacterial biomass). The KA, LA, and SR also soils exhibited lower oxidase (mean, 3.4–3.8-fold lower) and ^125^I^−^ uptake activity (mean, 2.7-fold lower) than the NT and FU soils. Finally, the inhibitory effects of autoclaving, bronopol and sodium azide on ^125^I^−^ uptake activity acted similarly amongst the KA, LA, and SR soils (Table 4). Autoclaving and sodium azide treatments were less effective as inhibitors of ^125^I^−^ uptake in FU soils, while bronopol and sodium azide were less effective in NT soils.

Based on the inhibitor results, it is reasonable to hypothesize that FU and NT soils exhibit enhanced ^125^I^−^ uptake (relative to KA, LA, and SR soils) for different reasons. Non-biological processes, such I^−^ sorption onto positively charged surfaces of Mn-, Al- or Fe-oxyhydroxides or clay minerals (Whitehead, 1974; Shetaya et al., 2012; Miller et al., 2015; Duborská et al., 2019) or I^−^ oxidation catalyzed by Mn-, Al- or Fe-oxyhydroxides (Fox et al., 2009; Shetaya et al., 2012), could play a secondary role in our ^125^I^−^ uptake experiments, particularly in FU soils where ∼15 of ^125^I^−^ uptake activity (%^125^I^−^ uptake per mg of dry soil) remained post-autoclaving (Table 4). Unfortunately, we did not have enough material from several of the sites to determine metal-oxyhydroxide or clay content in all the soils. However, if this residual activity does represent abiotic processes in the FU soils, then it could be subtracted from the inhibitor values for cycloheximide, bronopol and sodium azide treatments in this soil, resulting in levels of inhibition similar to those of the KA, LA, and SR soils. Future work should investigate the utility of using of Mn-, Al- or Fe-oxyhydroxides, clay content, or other chemical/physical soil properties as additional explanatory variables for ^125^I^−^ uptake potential of soils.

The NT soils had the highest average actinobacterial biomass and the highest oxidase activity measured using L-DOPA at pH 7.0, but ^125^I^−^ uptake was less susceptible to inhibition by bronopol and sodium azide in these soils. Bronopol and sodium azide would both inhibit general microbial activity through 1) oxidation of thiol groups on proteins (Shepherd et al., 1988) and 2) inhibition of respiration (cytochrome oxidase poisoning) (Lichstein and Soule, 1944), respectively, but only sodium azide is recognized as a laccase inhibitor (Johannes and Majcherczyk, 2000). It is possible that stable, extracellular oxidases of actinobacterial origin (i.e., SLACs) which are relatively insensitive to sodium azide and active at higher pH (high L-DOPA oxidase activity in NT soils at pH 7.0) are abundant in NT soils and are active in ^125^I^−^ oxidation. This scenario is supported by the results of the regression analysis with and without the NT samples. Without the NT samples, soil %N explained 93% of the variance in ^125^I^−^ uptake by the forest surface soils, while soil %N only explained ∼60% of the variance for the full dataset. Amongst all the other measurements examined, only actinobacterial biomass (adjusted *R* ^2^ = 0.652), and particularly, oxidase activity measured with L-DOPA (adjusted *R* ^2^ = 0.797) significantly improved the goodness-of-fit between soil %N and ^125^I^−^ uptake when added as a second explanatory variable (Supplementary Table S3).

The strong association between ^125^I^−^ uptake and %N content of the soils is notable. This association could reflect the link between %N content and microbial biomass/activity, however, %N was correlated to a much higher extent with ^125^I^−^ uptake than %C content or measures of microbial biomass, other than actinobacterial biomass (Figures 2, 3). Additionally, oxidase activity was correlated similarly with %N and %C, suggesting that the %N-^125^I^−^ uptake correlations were not simply a reflection of increased oxidase activity. One of the more favorable routes of soil incorporation into OM has been suggested to be through iodine binding to aromatic rings at the ortho/para position to the electron-donating group (e.g., amino group) of the aromatic ring (e.g., in proteins or humic acids) (Xu et al., 2012). Thus, the correlation between ^125^I^−^ uptake and N content might represent a combination of 1) the linkage between soil N content and soil microbial activity and 2) the availability of functional N groups in soil OM favoring covalent attachment of reactive iodine species. It should be noted that we did not observe a statistically significant correlation between C/N ratios and ^125^I^−^ uptake (*τ* = −0.17, *p* = 24.87).

Unlike sorption processes, where I^−^ binding under aerobic conditions decreases with increasing pH (Fukui et al., 1996; Kaplan et al., 2000; Söderlund et al., 2017), a correlation between soil pH and ^125^I^−^ uptake was not observed in the current study. The sum of biotic/abiotic processes promoting org-I formation in these temperate forest soils were independent of pH between 3.9 and 6.0. This does not imply, however, that individual processes were not pH dependent. For example, autoclaving had the least inhibitory effect on the FU soil (FUC10) with the lowest pH—where I^−^ binding to mineral surfaces would be stronger. Additionally, pH is expected to impact activity of potential iodide-oxidizing enzymes, with fungal laccases performing better at lower pH (3–6) and bacterial laccases, including SLACs, exhibiting maximal activity in neutral and basic soils. It would be interesting to further delineate the role of different laccase classes in soil iodination across different soils and ecosystems.

Soil C% and N% followed the expected pattern with depth, both were lower (4.6- and 3.9-fold lower, respectively; near significant correlation for both) in the 10–20 cm samples, and although, not significantly correlated with soil depth, ^125^I^−^ uptake also decreased with depth (1.7-fold less, on average). It has been suggested that soil, rather than vegetation, litterfall or humus, serves as the primary long-term reservoir of iodine in forest ecosystems (Roulier et al., 2019). Since iodine can be released from OM *via* degradation and under anaerobic conditions (Muramatsu et al., 1990; Shimamoto et al., 2011; Thiry et al., 2022), it is germane that microbial processes exist, down to at least 10-20 cm in the soil column of temperate forests, capable of promoting soil iodination under aerobic conditions. In the top layers of forest floors, iodination preferentially occurs with low molecular weight OM, some portion of which could be highly mobile as dissolved or colloidal particles (Xu et al., 2011; Söderlund et al., 2017; Roulier et al., 2022). As the OM ages and moves downward in the soil profile, higher molecular weight species or OM protected/bound to mineral surfaces will constitute a greater fraction of the total, making it more probable that free I^−^ would bind to these less-mobile, more refractory species. Therefore, as iodine moves down the soil column, even in inorganic form, its mobility may decrease, given there is sufficient OM available for binding and aerobic conditions persist. This scenario is consistent with the observation in many forests that the depth profile of iodine concentration in soils is not simply a function of C content, decreasing with depth, but rather iodine concentrations peak 5–40 cm below the surface (Xu et al., 2016; Roulier et al., 2018; Yang et al., 2019; Epp et al., 2020; Pisarek et al., 2022). In contrast to studies that have found differences in soil and humus iodine concentrations as a function of forest type (Roulier et al., 2019; Pisarek et al., 2022), we did not detect a difference in ^125^I^−^ uptake activity in soils collected under deciduous or coniferous trees.

## 6 Conclusion

The results from the current study provide compelling evidence that extracellular oxidases, most likely of bacterial origin, catalyzed soil iodination in forest soil slurries amended with ^125^I^−^ in short-term assays (12 h). Autoclaving and treating soils with antimicrobials and enzyme inhibitors (e.g., bronopol and sodium azide) significantly inhibited ^125^I^−^ uptake by soils, establishing that microbial processes were responsible for the majority of ^125^I^−^ uptake in both deciduous and coniferous, temperate forest soils collected from two depths (0–10 cm, 10–20 cm) at five sites on two continents. Two lines of evidence indicate that extracellular oxidases are the primary agents catalyzing ^125^I^−^ uptake by the forest soils. First, soil treatments with sodium azide led to a significantly greater reduction in ^125^I^−^ soil uptake than treatments with the antimicrobial agents, bronopol or cycloheximide (mean inhibition of 81%, 61% and 9%, respectively). Second, linear regression analysis showed a stronger association between ^125^I^−^ uptake and soil oxidase activity than between ^125^I^−^ soil uptake and any measure of microbial biomass evaluated or rates of soil respiration (*R* ^2^ for basal or glucose-stimulated rates of soil respiration vs. ^125^I^−^ soil uptake was <0.035, data not shown). For the first time soil iodide uptake activity was associated with a specific microbial group, the actinobacteria, whereas fungi appeared to play lesser role in iodide uptake by these soils. The expansive geographic range, inclusion of different forest types and soil depths in this study demonstrates that the observational correlation between laccase activity and iodine uptake in previous studies (Seki et al., 2013; Nihei et al., 2018) is common and widespread in forest soils. Going forward it is possible that measures of soil N content and soil oxidase activity (L-DOPA, pH 7) could be used in tandem to broadly predict iodide uptake capacity of different surface soil samples. In cases of high clay or metal oxyhydroxide content, especially in oligotrophic soils, it may be necessary to include terms accounting for these factors.

## Data Availability

The original contributions presented in the study are included in the article/Supplementary Material, further inquiries can be directed to the corresponding author.
