# Supplementary figures and images for: Iodide uptake by forest soils is principally related to the activity of extracellular oxidases
Source: Front Chem. 2023 Mar 2;11:1105641. doi: 10.3389/fchem.2023.1105641 (PMC10019592; doi:10.3389/fchem.2023.1105641)

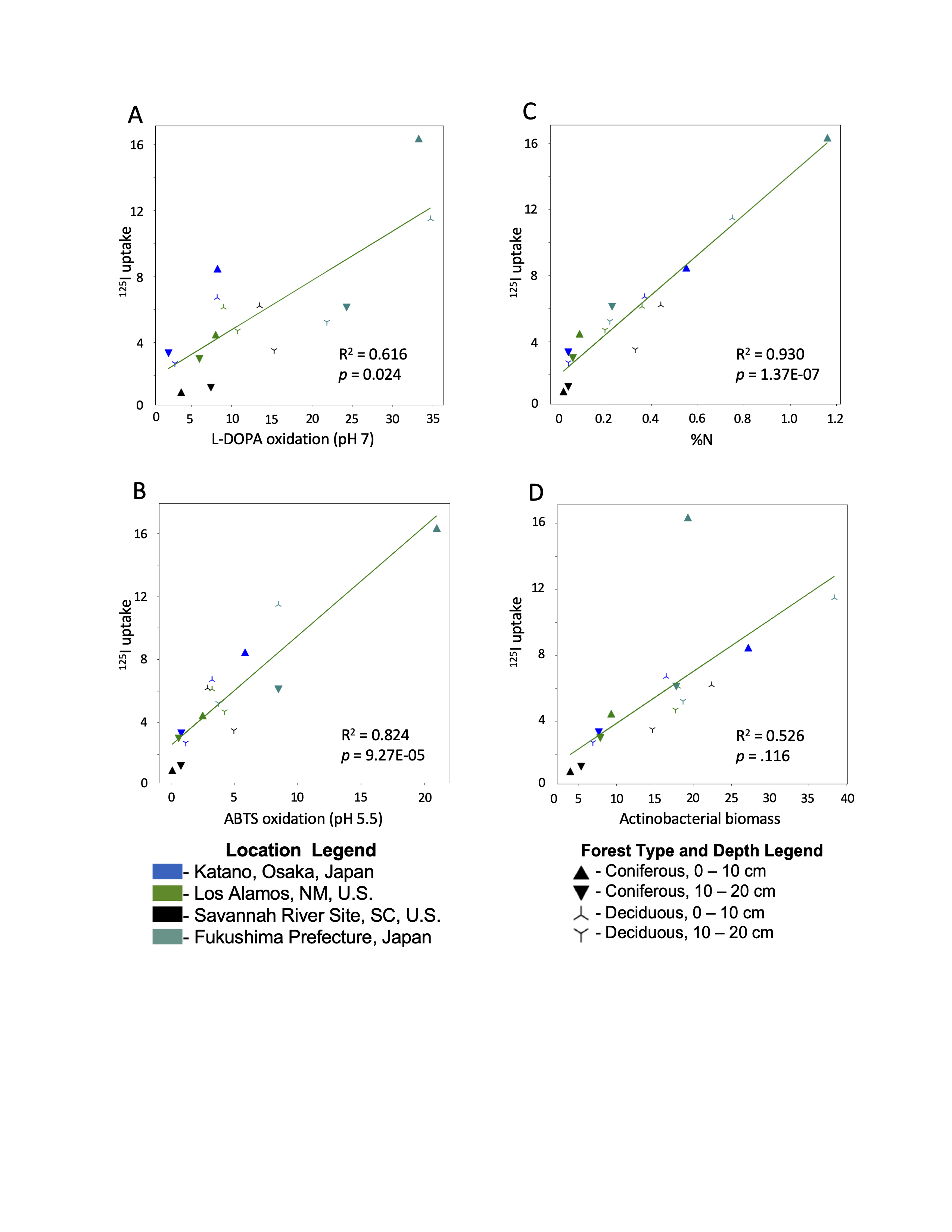

Supplement: Supplementary file 2 [file Image1.TIFF]

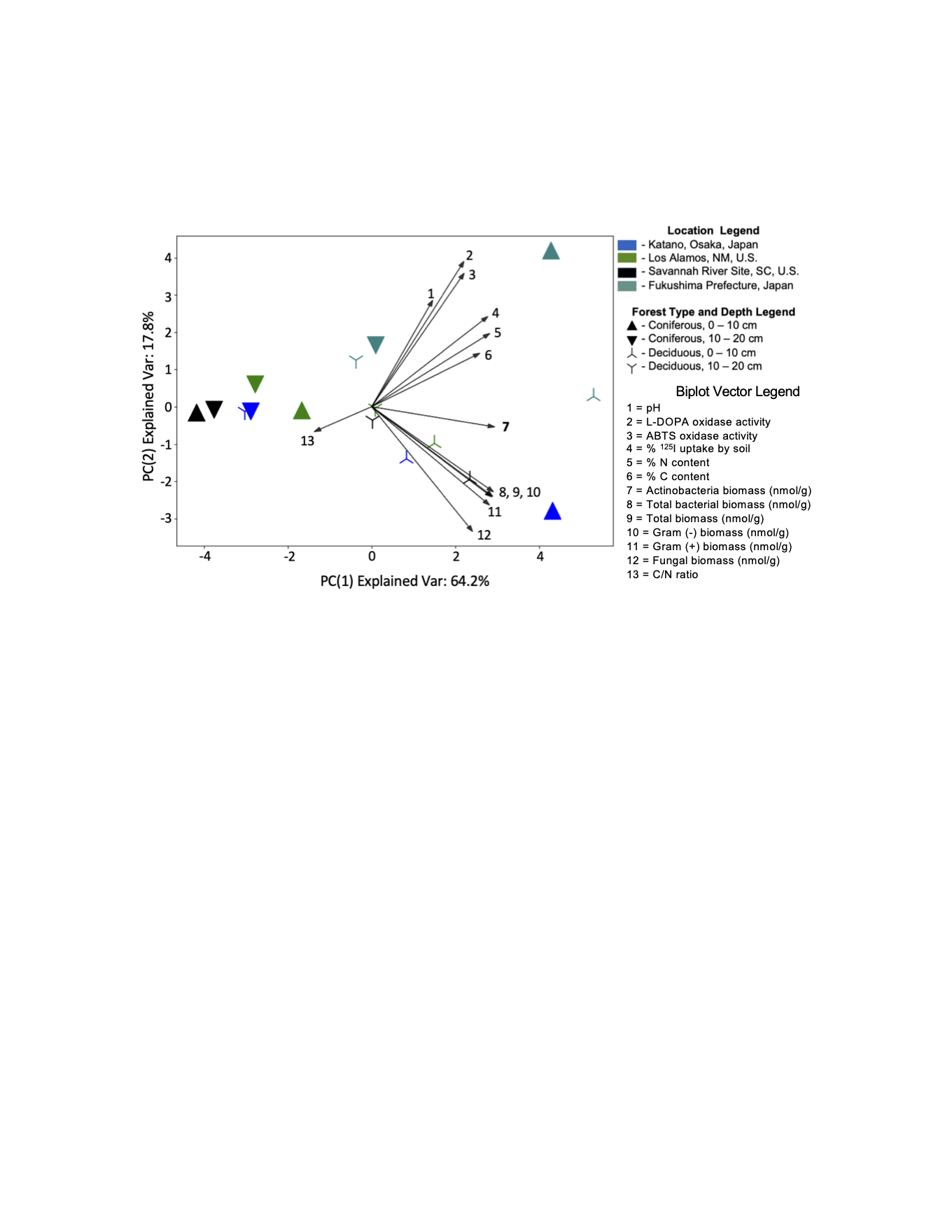

Supplement: Supplementary file 5 [file Image2.tiff]
