# Supplementary material for: Iodide uptake by forest soils is principally related to the activity of extracellular oxidases
Source: Front Chem. 2023 Mar 2;11:1105641. doi: 10.3389/fchem.2023.1105641 (PMC10019592; doi:10.3389/fchem.2023.1105641)
Supplement: Supplementary file 6 [file DataSheet1.docx]

Supplemental Figure 1. ^125^I uptake from forest soils correlated with (A) L-DOPA oxidation (pH 7.0), (B) ABTS oxidation (pH 5.5), (C) soil %N, (D) actinobacterial biomass. Oxidase activity units are reported as µmol h^-1^ g^-1^ dry soil. Actinobacterial biomass reported as nmol PLFAs g^-1^ dry soil. Site locations are designated by color: NT, red; KA, blue; LA, green; SR, black; FU, teal. Coniferous 0 – 10 cm, right side up triangle; Coniferous 10 – 20 cm, upside down triangle; Deciduous 0 – 10 cm, right side up star; Deciduous 10 – 20 cm, upside down star. ***Samples from site NT were excluded from this analysis (Figure 3 shows regression with full dataset)***.

Supplemental Figure 2. Actinobacteria biomass contributes to sample variance in a distinct manner. Two-component principal component analysis was performed on the standardized, non-redundant, numerical features of the samples. The resulting model explained 82.0% of variance in the dataset, with the majority of the explained variance contained within principal component 1. Samples with high iodine uptake aggregate in the upper right corner while samples from coniferous forests tend to aggregate towards the left side of the plot along PC1. The contributions of each property to the PCA model is represented in the biplot. Highly correlated features result in near-parallel vectors as seen with the non-Actinomyces biomass vectors (vectors 8-12) and chemical properties vectors (vectors 1-6) clustering together. Actinomyces biomass, vector 7, contributes almost exclusively to the most informative principal component and does not cluster with any other property. Similarly, the C/N ratio vector (vector 13) is distinct and may explain the aggregation of coniferous samples towards the left of the plot. ***Samples from site NT were excluded from this analysis (Figure 4 shows regression with full dataset)***.
